# A FRET-Based High Throughput Screening Assay to Identify Inhibitors of Anthrax Protective Antigen Binding to Capillary Morphogenesis Gene 2 Protein

**DOI:** 10.1371/journal.pone.0039911

**Published:** 2012-06-29

**Authors:** Michael S. Rogers, Lorna M. Cryan, Kaiane A. Habeshian, Lauren Bazinet, Thomas P. Caldwell, P. Christine Ackroyd, Kenneth A. Christensen

**Affiliations:** 1 Department of Surgery, Vascular Biology Program, Children's Hospital Boston, Harvard Medical School, Boston, Massachusetts, United States of America; 2 Department of Chemistry, Clemson University, Clemson, South Carolina, United States of America; University of Giessen Lung Center, Germany

## Abstract

Anti-angiogenic therapies are effective for the treatment of cancer, a variety of ocular diseases, and have potential benefits in cardiovascular disease, arthritis, and psoriasis. We have previously shown that anthrax protective antigen (PA), a non-pathogenic component of anthrax toxin, is an inhibitor of angiogenesis, apparently as a result of interaction with the cell surface receptors capillary morphogenesis gene 2 (CMG2) protein and tumor endothelial marker 8 (TEM8). Hence, molecules that bind the anthrax toxin receptors may be effective to slow or halt pathological vascular growth. Here we describe development and testing of an effective homogeneous steady-state fluorescence resonance energy transfer (FRET) high throughput screening assay designed to identify molecules that inhibit binding of PA to CMG2. Molecules identified in the screen can serve as potential lead compounds for the development of anti-angiogenic and anti-anthrax therapies. The assay to screen for inhibitors of this protein–protein interaction is sensitive and robust, with observed Z' values as high as 0.92. Preliminary screens conducted with a library of known bioactive compounds identified tannic acid and cisplatin as inhibitors of the PA-CMG2 interaction. We have confirmed that tannic acid both binds CMG2 and has anti-endothelial properties. In contrast, cisplatin appears to inhibit PA-CMG2 interaction by binding both PA and CMG2, and observed cisplatin anti-angiogenic effects are not mediated by interaction with CMG2. This work represents the first reported high throughput screening assay targeting CMG2 to identify possible inhibitors of both angiogenesis and anthrax intoxication.

## Introduction

Angiogenesis is the process of blood vessel formation that occurs when new capillaries sprout from pre-existing vessels [1]. It is a biological process that is normally only seen in the female reproductive system, in fetal development, and in wound healing [1]–[4]. Angiogenesis is required for any process that results in the accumulation of more than a few microns of new tissue, as well as many processes involving tissue remodeling. As such, it is a characteristic of multiple common disease pathologies that involve inappropriate tissue development [5], including cancer [6], [7], cardiovascular disease, arthritis, psoriasis, several rare genetic diseases [8], and a variety of eye disorders, including macular degeneration [9], diabetic retinopathy [10], herpetic keratitis, trachoma, and retinopathy of prematurity [11]. Therapies that target angiogenesis can thus be used to halt or slow the development of these disorders, and have been shown to be effective in a variety of diseases [12]–[15].

We have previously demonstrated that protective antigen (PA), a non-pathogenic component of the anthrax toxin which binds to endothelial cell surface receptors, can inhibit angiogenesis [16]. Treatment with a PA mutant (PA^SSSR^), with three altered amino acids [17], increased inhibition of vessel growth in both VEGF-and bFGF-induced corneal neovascularization assays, inhibited migration of endothelial cells, and resulted in pronounced (≥40%) reductions in tumor growth [16]. Anthrax toxin binds and co-opts two endothelial cell surface receptors, anthrax toxin receptor 1 (ANTXR1; also called tumor endothelial marker 8, TEM8) [18], and anthrax toxin receptor 2 (ANTXR2; also called capillary morphogenesis gene 2 protein, CMG2) [19]. Significantly, PA mutants that do not bind these receptors do not inhibit angiogenesis, and the binding affinity of individual PA mutants for the receptors correlates with their degree of inhibition [16]. These data strongly suggest that interaction with an anthrax receptor is responsible for the anti-angiogenic effects of PA^SSSR^.

The normal biological function(s) of TEM8 and CMG2 have not been fully described, although the existing data indicates that these receptors are involved in angiogenic processes, consistent with the observed impact of PA^SSSR^ binding on angiogenesis. Both receptors contain a von Willebrand A or integrin-like inserted I domain, with 60% identity in this region, and are the closest related proteins to integrins, which are involved in cell binding to a variety of extracellular matrix components. TEM8 was initially identified as a protein expressed on colon tumor endothelium, but not on normal endothelial cells [20], and was subsequently detected in a variety of angiogenic or cancerous endothelial cell types [21], [22]. TEM8 knockout mice demonstrate alterations in extracellular matrix deposition, and changes in the growth rate of specific tumors [23]. Importantly, TEM8 expression is upregulated in tumor-associated endothelial cells, and receptor expression is linked to disease progression in several cancer types [22], [24], [25]. Protein overexpression and gene knockdown experiments demonstrate that TEM8 is involved in endothelial cell migration and tube formation [26] via interactions with the extracellular cellular matrix component collagen a3(VI) [27], and linkage to the actin cytoskeleton [28]. Finally, TEM8-specific antibodies strongly inhibit the growth of a variety of solid tumors, but have no effect on either the matrigel plug angiogenesis assay, or on wound healing, suggesting some tumor specificity in TEM8 expression [29]. CMG2 is similarly involved in antiangiogenic processes. The receptor was initially identified as the product of the capillary morphogenesis gene 2, which is upregulated in endothelial cells during capillary formation in collagen gels [30]. CMG2 binds both laminin and collagen type IV [30], suggesting that like TEM8, this receptor's physiological role involves interactions with the extracellular matrix that are required for angiogenesis. Indeed, the receptor is highly expressed in both normal and cancerous vasculature, and its pattern of expression colocalizes with collagen type IV [31]. Genetic mutations in CMG2 result in the related disorders juvenile hyaline fibromatosis and infantile systemic hyalinosis [32] that are characterized by multiple recurring tumors and inappropriate deposition of hyalin, an extracellular matrix material. Like TEM8 knockout mice, female mice which lack the CMG2 receptor do not give birth, an effect apparently mediated by defects in uterine extracellular matrix remodeling [33]–[35]. Importantly, venous endothelial cells that overexpress CMG2 show increased proliferation and formation of capillary-like networks, while CMG2 knockdown cells demonstrate significantly impaired endothelial cell proliferation [31]. Together, the TEM8 and CMG2 data suggest that binding of endogenous ligands to anthrax toxin receptors is involved in angiogenic processes *in vivo*, and that inhibition of these interactions by competing ligands should inhibit vascular growth. Hence, ANTXR-targeted small molecule angiogenesis inhibitors represent a new strategy for anti-angiogenic therapy.

Inhibition of the receptor-ligand interaction could also be used as an anthrax therapy. Anthrax intoxication begins when PA binds to CMG2 or TEM8 on the cell surface, prompting cleavage of PA by cell-surface proteases [36], oligomerization to a heptameric species, complex formation with the pathogenic toxin components, Lethal Factor (LF) and Edema Factor (EF) [37], and their subsequent delivery to the interior of the cell via endocytosis [38]–[40]. The low pH of the mature endosome then prompts toxin rearrangement and translocation of EF and LF to the cytosol [41]. Since receptor binding is the first step in this process, inhibition of receptor binding is a plausible treatment for anthrax intoxication, and small molecules that block the interaction(s) of PA with its receptors would be effective anthrax toxin inhibitors. Previous attempts to generate anthrax therapies [42]–[56] have targeted several individual steps in the process of anthrax endocytosis, but only a single study addresses receptor inhibition via receptor binding [57]. Importantly, unlike other proposed therapies, receptor-targeted therapies can circumvent the traditional difficulty of testing anthrax inhibitors with active toxin *in vivo*, because compounds could be initially tested for their safety and cytotoxicity in the context of ocular or cancer related angiogenesis.

We have developed a high throughput screening assay to identify potential inhibitors of the interaction between PA and the ATR2 (CMG2) receptor. The assay is based on fluorescence or Förster resonance energy transfer (FRET) observed upon interaction of dye-labeled PA and a dye-labeled CMG2 truncation. Steady-state FRET-based assays like this are ideally suited for high throughput screening (HTS) methodology because they are simple, sensitive, and easily automated. When conducted ratiometrically, these measurements yield a quantitative readout of the macromolecular association state that is corrected for experimental fluctuations occurring between or across well-plates, including differences in excitation power, pathlength, and photobleaching. Here we demonstrate a ratiometric steady-state FRET-based screening assay that is highly effective and capable of identifying potential PA-CMG2 inhibitors. The anti-angiogenic effects of compounds identified from preliminary screens of small molecule libraries are characterized and described.

## Results

### FRET screening assay design

FRET is the highly distant-dependent through-space transfer of energy from a fluorescent donor to an acceptor molecule. FRET between dye-labeled molecules occurs in cases when the donor dye's fluorescence emission spectrum overlaps with an acceptor dye's excitation spectrum. In this case, when the fluorescence of the donor molecule is excited directly, a portion of that excitation energy can be funneled to the acceptor molecule instead of being followed solely by emission at the characteristic emission wavelength of the donor dye. What is observed, instead, is decreased emission of the donor, accompanied by increased (or sensitized) emission for the second, acceptor, dye. Importantly, such energy transfer can only occur when the two dyes are in close spatial proximity (typically less than 100 Å). Hence, FRET is a sensitive probe of macromolecular association. FRET has been used previously to measure the kinetics and stoichiometry of PA binding to a soluble truncation of the extracellular domain of CMG2 [58]. Here we describe an adaptation of this assay for high throughput screening.

**Figure 1:**
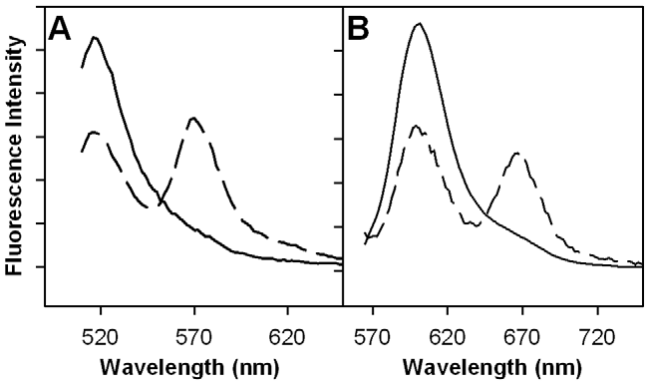
Fluorescence emission spectra of labeled protein reagents exhibit resonance energy transfer. **A**) Fluorescence emission spectra of 10 nM PA^E733C*AF488^ (solid line; donor alone) and 10 nM PA^E733C*AF488^ +10 nM CMG2^R40C*AF546^ (dashed line; donor + acceptor) in HBST. Both spectra were acquired using 485 nm excitation. **B**) 10 nM PA^E733C*AF568^ (solid line; donor alone) and 10 nM PA^E733C*AF568^ +10 nM CMG2^R40C*AF647^ (dashed line; donor + acceptor) in HBST. Both spectra were acquired using 556 nm excitation.

Using directly labeled protein reagents in a simple homogeneous assay format, we have developed a nearly ideal high throughput FRET screening assay to identify small molecules and natural product extracts that inhibit the PA-CMG2 protein-protein interaction. As with other FRET assays, the screening assay uses interactions between fluorescent labels on the two proteins to report binding. We expressed, purified, and covalently labeled a single cysteine PA mutant, PA^E733C^, with an Alexa Fluor 488 label (AF488; λ_ex_  = 488 nm; λ_em_  = 525 nm). This labeled PA was designed to be the FRET donor. A truncated soluble version of CMG2 consisting of amino acid residues 40–217 containing the mutations R40C and C178A (CMG2^R40C^ [58]) was also expressed, purified, and covalently labeled with Alexa Fluor 546 (AF546; λ_ex_  = 546 nm; λ_em_  = 570 nm). This labeled CMG2 was designed to be the FRET acceptor. To show energy transfer between dye-labeled PA and CMG2, we made an equimolar mixture (10 nM) of the two labeled proteins that was excited at 485 nm, which predominantly excites PA^E733C*AF488^, the donor molecule. Specific spectral changes relative to free protein were observed under these conditions. Figure 1A shows the emission spectrum of 10 nM PA^E733C*AF488^ alone (solid line), overlaid with the emission spectrum of an equimolar mixture of the PA^E733C*AF488^ and CMG2^R40C*AF546^ (dashed line). A clear decrease in PA^E733C*AF488^ emission intensity was observed at ∼ 525 nm (donor quenching) for the mixture, together with enhancement of the emission intensity of the CMG2^R40C*AF546^ acceptor dye at ∼570 nm (sensitized emission). These significant spectral changes strongly suggest energy transfer between the dyes, reflecting PA-CMG2 binding. Rather than simply using either the decreased donor emission or the increased acceptor emission to assess the degree of binding, we have used a ratio of their values (i.e. I_acceptor emission_/I_donor emission_  = I_AF546_/I_AF488_) to gauge binding; the ratio corrects for instrumental fluctuations and helps minimize possible systematic errors in the fluorescence measurements.

Prior to screening, we were concerned about potential photoluminescence or autofluorescence of compounds in the screening library. Hence, we also tested the protein reagents labeled with an alternate red-shifted dye-pair, in case we observed competing luminescence from significant numbers of library compounds using 485 nm excitation. Similar to the results for the AF488/AF546 dye-pair labeled proteins, we observed significant energy transfer in the observed fluorescence emission spectra when 10 nM PA^E733C*AF568^ and CMG2^R40C*AF647^ were mixed (Figure 1B). Again, we observed a clear decrease in intensity of AF568 dye-labeled PA donor emission intensity at ∼ 605 nm (donor quenching) and enhancement of the intensity of AF647 dye-labeled CMG2 acceptor at ∼675 nm (sensitized emission) was observed. Slightly less energy transfer was observed for AF568/AF647 dye-pair relative to the original AF488/AF546 dye-pair, presumably due to differences in the relative Förster distances for the two dye pairs. Only a small percentage of the tested library compounds had sufficient luminescence to interfere with assay performance in the pilot screens reported here; therefore, we used the slightly better performing AF488/AF546 dye pair in all subsequent screening experiments. We expect that this alternate dye pair could be used to achieve excellent assay performance in conditions where compounds have significant short wavelength background fluorescence.

The goal of our high throughput screen was to identify compounds that inhibit the PA-CMG2 interaction. Because the observed FRET is sensitive to the PA-CMG2 interaction, it is dramatically reduced in the presence of compounds that inhibit the interaction. For example, addition of EDTA, which shifts the K_d_ by orders of magnitude [58], causes a two-fold reduction in the fluorescence emission ratio (R = I_AF488-PA_/I_AF546-CMG2_;/R = I_590 nm_/I_535 nm_), reflecting substantially reduced PA-CMG2 binding under the conditions of the validation assay (see below). This reduction in FRET ratio in the presence of inhibitors became the basis for the subsequent high throughput assay. Using the AF488/AF546 dye pair described above, we measured the FRET ratio for PA^E733C*AF488^ and CMG2^R40C*AF546^ in the presence of possible inhibitors, and compared that value to that for labeled PA and CMG2 alone. Significant reductions in observed FRET ratio were then used to infer inhibition of the PA-CMG2 interaction by individual library compounds.

More specifically, the high throughput screening assay measures the FRET between CMG2 and PA in 384 well-plates in the presence of potential inhibitors. Aliquots of CMG2 were added to individual wells, followed by addition of small volumes of test compound, incubation to allow possible interaction of the test compound with CMG2, and addition of an aliquot of PA. After further incubation, the fluorescence intensities at 535 nm (AF488-labeled PA donor) and 595 nm (AF546-labeled CMG2 acceptor) were read for each well. The ratio of these values (F_595_/F_535_) was calculated for each well and compared to the corresponding ratio for the negative control (no added compound) and positive control (EDTA added with the CMG2 solution) on each well-plate. To correct for possible day to day or plate to plate variation, a value for the fraction inhibition could also be calculated relative to the controls on each plate (I_norm_ = (R_obs_-R_neg_)/(R_pos_-R_neg_); this value reflects a normalized value for inhibition of the PA-CMG2 interaction. In this case, zero normalized inhibition reflects no inhibition (equal to the negative control) and a normalized inhibition value of 1 reflects complete inhibition (equal to the EDTA positive control). To minimize use of library compounds and the possibility of false positives, potential inhibitors were tested in duplicate (*i.e*. were added to each of two wells). To determine whether individual compounds qualified as “hits”, the observed FRET ratios (F595/F535) were averaged for all wells without EDTA in a given plate, and the standard deviation of this mean was calculated. Based on this standard deviation (*s_F595/F535_*), an arbitrary cutoff FRET ratio was assigned that was three standard deviations (i.e. 3*s_F595/F535_) lower than the negative control. When both wells of an individual compound had FRET ratios below this cutoff, the compound was assigned as a “hit”. This cutoff assignment could also be performed using a normalized fraction inhibition rather than absolute FRET ratios.

### High throughput screen optimization and validation

Within the context of the high throughput screen described above, it was necessary to adjust experimental conditions to optimize assay performance. The overall performance of a screening assay is related to multiple parameters that include stability, sensitivity, reproducibility, robustness, and well-to-well variation across the screening plate. Most of these parameters affect the observed Z' value, which is calculated from the standard deviation of the positive and negative control wells.[59] As a result, the Z' value can be used to assess assay performance, based on measurements of the positive and negative controls alone. As described above, the negative control was assigned as the observed FRET ratio in the absence of any inhibitor, while the positive control was assigned as the observed FRET ratio in the presence of EDTA, a potent inhibitor of CMG2 binding. Generally, a Z'>0.5 is the minimum acceptable value for an interpretable screening assay while Z'>0.7 is considered a good result. Higher Z'-values are even better. During the optimization and validation phases of development, assay solution and acquisition parameters were adjusted to optimize Z'.

First, the concentration of the labeled proteins was adjusted to minimize reagent consumption and any inner-filter effect(s), while maximizing signal intensity. High signal-to-noise measurements minimize instrument noise and decrease standard deviations to improve Z'. The optimal concentrations were found to be 7.5 nM PA^E733C*AF488^ and 13 nM CMG2^R40C*AF546^. Given the high affinity of the PA-CMG2 interactions (K_d_≥170 pM [58]), these concentrations are consistent with quantitative binding of CMG2 to PA in the absence of inhibitor.

**Figure 2:**
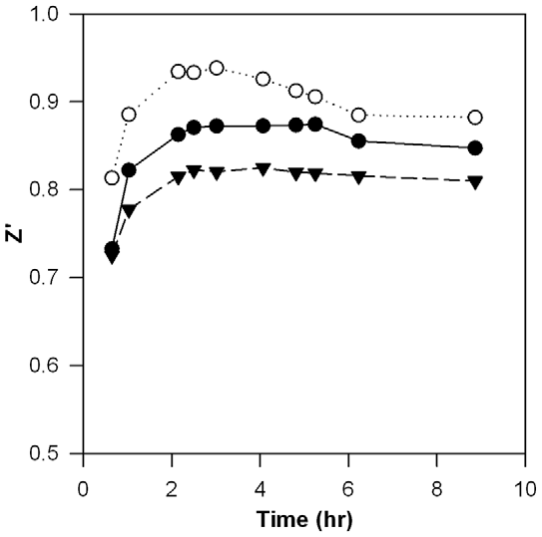
Time course of measured Z '**.** Z' was measured over time in polystyrene (•), polypropylene (▾), and commercially available low-binding (○) 384 well-plates. All wells contained 7.5 nM PA^E733C*AF488^ and 13 nM CMG2^R40C*AF546^ in HBST plus either 5 mM NaCl (negative control; 168 wells) or 5 mM EDTA (positive control; inhibitor; 168 wells).

We monitored Z' as a function of PA-CMG2 equilibration time to ensure that the equilibration time was sufficient to achieve optimal assay performance. These experiments revealed that initial experimental conditions resulted in a time-dependent decrease in Z', as well as Z' values below acceptable limits (data not shown). We suspected that since we were using very dilute protein solutions in the assay, non-specific binding of the labeled protein reagents onto the well-plate was the cause of the observed decrease in Z'. Consistent with this hypothesis, a stable Z' was observed when the experiment was repeated with the addition of 0.1 mg/ml BSA or 0.075% (v/v) Tween-20 to the CMG2 solution. Addition of Tween-20 may have an additional beneficial effect, since aggregating nonspecific compounds can create false “hits” during screening that can be ameliorated by addition of a detergent [60]. As a result, Tween-20 was included in all subsequent experiments. Figure 2 shows Z' as function of PA-CMG2 equilibration time in the presence of Tween-20. These data indicate that optimal assay performance is achieved with a 2–4 hour incubation, presumably reflecting complete binding of PA to CMG2 in this time frame. This observation is consistent with the published association kinetics in this system (k_a_  = 1.1 x 10^5^ M^−1^s^−1^ [58]), which predict complete binding of CMG2 by PA within this time frame at these protein concentrations. PA was incubated with CMG2 for 4 hours prior to fluorescence measurement in all subsequent validation and pilot screening assays.

As part of our analysis of the effects of incubation time on assay performance, we also tested the use of different well-plate materials in the presence of Tween-20. We observed different assay performance in polystyrene, polypropylene, and specialized polypropylene plates designed to reduce nonspecific binding to well surfaces (Figure 2). These data indicate that the material composition of the well-plates affects assay performance, even in the presence of Tween-20. Although the specialized low-binding polypropylene plates produced the highest Z' value, their higher cost was prohibitive in our situation, and all subsequent screens were carried out with polystyrene well-plates, the next best performer.

As library compounds were to be added in DMSO solutions, we also evaluated the effect of DMSO on assay performance. While addition of 10% of the total assay volume of DMSO had a significant but small effect on the assay (10% reduction in Z'), a 1% DMSO addition had no measureable effect on Z', and was used in the subsequent pilot and library screenings.

These data were used to develop an optimized screening assay protocol (see Materials and Methods). Specifically, a CMG2 solution containing Tween-20 was added to individual wells of polystyrene well-plates, followed by pin transfer of small volumes of library compounds in volumes sufficiently small to keep total DMSO concentrations low (<1% v/v) and an equilibration designed to allow interaction of potential inhibitors with CMG2 prior to addition of PA. Addition of PA solution to each well was followed by a 4 hour incubation of PA with CMG2. Delivered concentrations of CMG2 and PA were chosen to ensure that their final concentrations in solution reached optimal levels. Negative controls (no added library compound) and positive controls (EDTA added to the CMG2 solution to prevent PA binding) were present on each plate. Comparison of measured FRET ratios for inhibitor and control wells was then used to assess possible inhibition of PA-CMG2 binding by library compounds.

**Figure 3:**
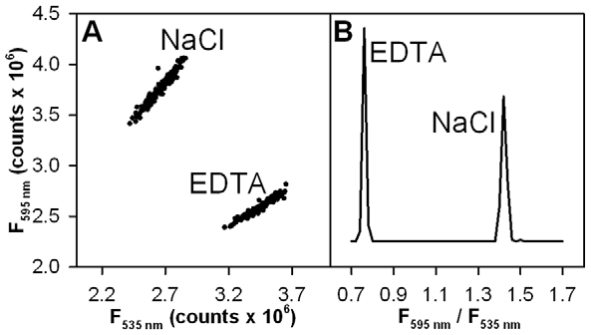
PA-CMG2 high throughput screening assay performance. A polystyrene 384 well-plate filled with 7.5 nM PA^E733C*AF488^ and 13 nM CMG2^R40C*AF546^ in HBST plus either 5 mM NaCl (negative control; 168 wells) or 5 mM EDTA (positive control; inhibitor; 168 wells). **A**) Scatter plot of donor (λ_em_  = 535 nm) and acceptor (λ_em_  = 595 nm) emission showing the shift induced by addition of EDTA (positive inhibitor control). **B**) Histogram of the F_595 nm_/F_535 nm_ ratio taken from the same data. For these data Z' = 0.91.

These optimized conditions were used for validation of the PA-CMG2 screening assay. In this validation assay, half of the wells in each plate were assigned to positive control conditions, while the other half were negative controls. These data are shown in Figure 3. Two well-separated tight clusters of data are observed; one for the positive control, and one for the negative control. These same data are shown in a histogram format in Figure 3B, expressed as a ratio of the fluorescence intensities. These data demonstrate that binding and inhibition are extremely well separated in our screen, a result which promotes sensitive detection of binding inhibition. The validation assay results reflect outstanding assay performance, with measured Z' values consistently at or above 0.9.

### Pilot screening with the PA-CMG2 assay successfully identified CMG2 inhibitors with anti-angiogenic properties

**Figure 4:**
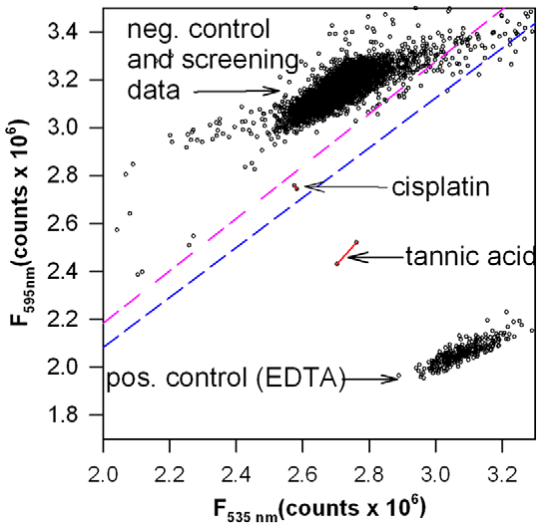
Results from screening a small library of known bioactives. Scatter plot of screening data for a small library of known bioactive small molecules using the PA-CMG2 screening assay. The pink dashed line shows the arbitrary cutoff representing inhibition greater than 3 standard deviations from the plate mean; a blue dashed line showing 5 standard deviations from the plate mean has also been drawn for comparison. Arrows point to compounds with inhibition values larger than the cutoff for both wells (red lines connect duplicate wells). Compounds that cluster in the top right hand corner have significant compound autofluorescence and were not considered potential hits.

Using the optimized and validated assay, we screened a small library of 2,640 known bioactive small molecules. The library was selected for structural diversity and includes many classes of compounds, including ion channel blockers, GPCR ligands, second messenger modulators, nuclear receptor ligands, actin and tubulin ligands, kinase inhibitors, protease inhibitors, gene regulation agents, lipid biosynthesis inhibitors, as well as other well-characterized compounds that perturb cell pathways. Approximately 50% of FDA-approved drugs are included in the library, including a significant subset of substances known to influence brain activity. Hence, the library was not biased towards anti-angiogenic or anti-cancer activity. Figure 4 shows data from the pilot screen of this library. Only two of the compound solutions tested, cisplatin and tannic acid, had a F_595 nm_/F_535 nm_ ratio greater than 3 standard deviations from the plate mean for both of the duplicate wells, indicating significant and repeatable inhibition of the PA-CMG2 interaction. Both solutions were analyzed with respect to inhibition of PA-CMG2 binding, disruption of angiogenesis, and interaction with CMG2.

**Figure 5:**
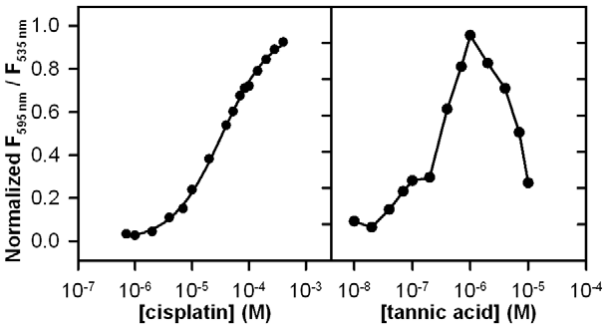
IC_50_ of cisplatin and tannic acid for PA-CMG2 interaction. **A**) IC_50_ was measured for cisplatin using the PA-CMG2 screening assay. Data were fit to the single site binding isotherm; **B**) IC_50_ was measured for tannic acid using the PA-CMG2 screening assay. Data could not be fit to any binding model. Ratios presented here are calculated differently than presented elsewhere in the paper (R_obs_  = F_535nm_/F_595nm_) in order to present a binding curve with standard appearance, and are normalized against the positive and negative control wells on each plate (R_norm_ = (R_obs_-R_neg_)/(R_pos_-R_neg_)).

**Figure 6:**
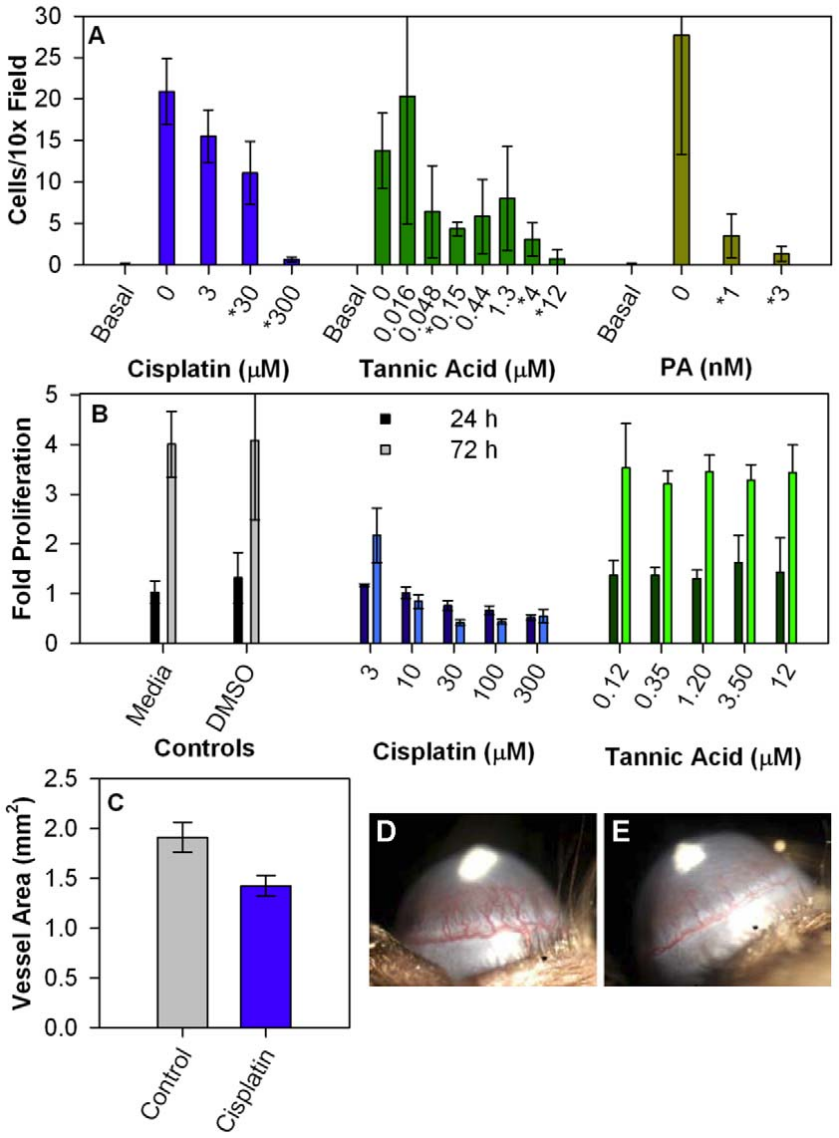
*In vitro and in vivo* effects of identified PA-CMG2 inhibitors. **A**) The effect of various concentrations of cisplatin or tannic acid on endothelial cell migration toward serum-containing medium; effects of PA administration are shown for comparison. Error bars represent standard deviation of the mean (SD; n = 12, 4 10X fields from 3 membranes). Statistically significant differences between inhibitor-containing and inhibitor-free conditions (as determined by the Holm–Bonferroni method) are shown by asterisks (*) preceding the concentration on the horizontal axis. Each of these experiments was repeated at least twice with concordant results. **B**) The effect of various concentrations of cisplatin and tannic acid on cell survival and proliferation. Proliferation assays were performed for 24 (dark bars) and 72 hours (light bars) in the indicated concentration of cisplatin and tannic acid and cell numbers assessed using Cyquant and normalized to controls fixed in ethanol at time 0. At 24 hours, the highest concentration of each molecule resulted in statistically significant decreases (as determined by t-test); however following Bonferroni correction, these differences were not significant. At 72 hours all cisplatin doses resulted in statistically significant decreases in cell number (as determined by the Holm–Bonferroni method). Error bars represent SD, n = 3 wells. For DMSO, 7 concentrations from 0.001% to 1% were assessed and no significant difference was observed among conditions. Therefore these data were pooled and thus for this sample n = 21 wells. No significant difference between DMSO-containing and media-only samples was observed (t-test). **C**) Effect of treatment with 4 mg/kg/day cisplatin on vessel area in the corneal micropocket assay. Control mice were treated with vehicle alone. Error bars represent SEM (n = 10 eyes). The observed difference is statistically significant (p<0.03 by t-test). No animal weight loss with cisplatin administration was observed. **D**) Representative image of control eye vessel area. **E**) Representative image of cisplatin treated mouse eye vessel area.

As shown in Figure 5A, cisplatin inhibits the PA-CMG2 interaction with an IC_50_ of 34 μM, measured using the FRET assay; the IC_50_ curve had a Hill coefficient indistinguishable from one, indicating that inhibition likely occurs by formation of a 1∶1 complex. Cisplatin also had statistically significant anti-angiogenic effects. Cisplatin inhibits cell migration in a dose-dependent manner (Figure 6A), and has a modest but statistically significant effect on corneal vessel growth (Figure 6C). However, since cisplatin is a known DNA cross-linker with established cytotoxicity [61], observed anti-angiogenic effects could result from mechanisms unrelated to CMG2 binding. Indeed, cell proliferation experiments designed to investigate cisplatin cytotoxicity showed statistically significant reductions in cell proliferation following long incubation with cisplatin (Figure 6B), suggesting the possibility that cytotoxicity could contribute to cisplatin's inhibition of cell migration and corneal vessel growth.

**Figure 7:**
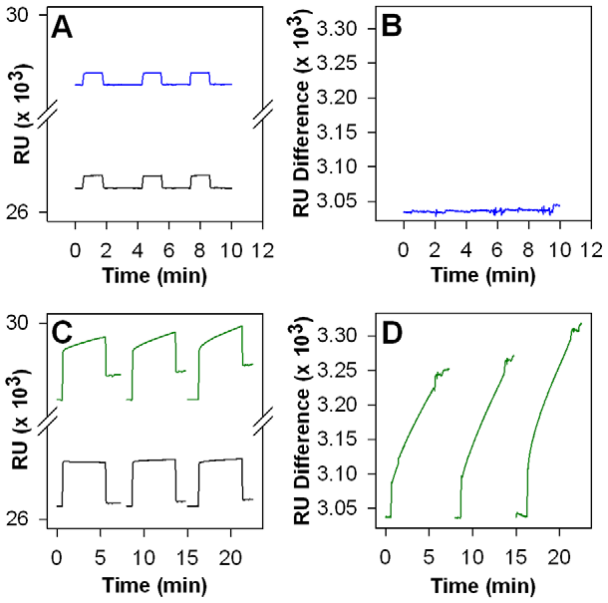
SPR analysis of cisplatin and tannic acid binding immobilized CMG2. **A**) SPR sensorgram using a streptavidin-modified carboxydextran gold sensorchip where biotinylated CMG2 was immobilized in channel 2 (blue trace) and biotin-PEG was immobilized in channel 1 (black trace) as a control. A solution of 500 μM cisplatin in HBST was flowed over the sensor surface. Three independent injections are recorded. **B**) A difference sensorgram is shown based on the data in Figure 7A. **C**) SPR sensorgram where biotinylated CMG2 was immobilized in channel 2 (green trace) and biotin-PEG was immobilized in channel 1 (black trace) as a control. A solution of 1 μM tannic acid in HBST was flowed over the sensor surface. Three independent injections are shown. **D**) A difference sensorgram is shown based on the data in Figure 7C.

**Figure 8:**
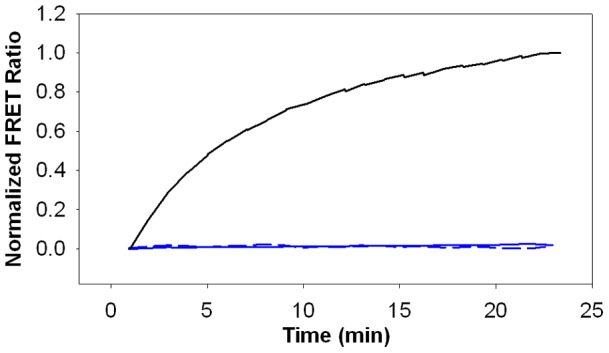
FRET kinetic assay for cisplatin binding to CMG2 and PA. Fluorescently labeled CMG2^R40^ **.** ^C C178A*AF546^ (1 μM) was preincubated with vehicle alone (DMSO) for 30 minutes before being rapidly diluted into PA^E733C*AF488^ to a final concentration of 10 nM of both proteins and the acceptor/donor fluorescence ratio was recorded over time (black line). Similarly, fluorescently labeled CMG2 was also preincubated with 500 μM cisplatin for 30 minutes before being rapidly diluted into PA as in the vehicle control. Fluorescence was again recorded over time (solid blue line). Fluorescent PA (1 μM) was also preincubated with 500 μM cisplatin for 30 minutes before being rapidly diluted into a solution of labeled CMG2. Fluorescence was recorded over time (dashed blue line). Excitation was 485 nm.

We carried out follow-up binding studies to provide support for direct interaction of cisplatin with CMG2. We could not detect cisplatin interaction with CMG2 by surface plasmon resonance (SPR) (Figures 7A and B). However, since cisplatin is a relatively small molecule (MW 300), and SPR detects intermolecular association via mass increases, a negative SPR result under these conditions does not completely rule out cisplatin binding. As a result, we also conducted a kinetic assay to investigate interaction of cisplatin with CMG2. In these experiments, labeled CMG2 was first preincubated with cisplatin at concentrations likely to result in significant interaction (i.e.10X the IC_50_ measured by FRET), then rapidly diluted with a solution of labeled PA to cisplatin concentrations at least 10-fold below the measured IC_50_. Under these conditions, any preformed cisplatin-CMG2 complex must dissociate before the FRET positive PA-CMG2 complex can form. As a result, reductions in the observed association rate of CMG2-PA in the presence of cisplatin provides evidence for interaction of cisplatin with CMG2. As shown in Figure 8, preincubation with cisplatin had a profound effect on CMG2-PA binding, indicating that the compound interacts with CMG2. However, similar kinetic experiments also indicate interaction of cisplatin with PA; preincubation of cisplatin with labeled PA before addition of excess labeled CMG2 resulted in nearly identical disruption of CMG2-PA binding. Hence, cisplatin is a nonspecific inhibitor of PA-CMG2 binding that interacts with both CMG2 and PA. Indeed, interaction of cisplatin with PA has been previously reported [62]. The inhibition appears irreversible for both proteins, as evidenced by the failure of both samples to show any association with an excess of the alternate protein after incubation with cisplatin (Figure 8). As cisplatin is a known cross-linker, it is possible that cisplatin inhibits the PA-CMG2 interaction via formation of covalent adducts. Thus, while cisplatin is not a viable therapeutic lead compound, its isolation demonstrates that the FRET high throughput screen was effective in identifying an effective inhibitor of PA-CMG2 interaction.

We also characterized the binding and anti-angiogenic properties of the tannic acid solution. We used the FRET assay to assess IC_50_ of tannic acid on the PA-CMG2 interaction. As shown in Figure 5B, the tannic acid IC_50_ curve was complex, suggesting multiple binding modes or the presence of impurities with a range of binding affinities. Due to the complex binding isotherm, we could not determine an IC_50_. However, these binding data indicate that there are concentrations of tannic acid that clearly inhibit PA-CMG2 interaction. Hence, isolation of tannic acid in the high throughput screen was not a false positive. Binding of the tannic acid solution to CMG2 was corroborated by SPR. As shown in Figures 7C and D, addition of aliquots of the tannic acid solution to CMG2 result in clear changes in SPR behavior relative to CMG2, versus control sensor surface. These data were not collected under conditions that allowed quantitative determination of binding affinity; however, the data indicate significant interaction of CMG2 with tannic acid or other solution components at a total concentration of 1 μM, suggesting dissociation constants similar to or lower than this value.

We assessed the anti-angiogenic effects of this tannic acid solution using the endothelial cell migration assay (Figure 6A) and observed statistically significant inhibition. This inhibition did not appear to reflect cytotoxicity, as no statistically significant effect on cell proliferation was observed over the range of concentrations that inhibited cell migration (Figure 6B). Hence, tannic acid, or impurities in this tannic acid solution, both bind CMG2 and inhibit angiogenesis. We also measured *in vivo* anti-angiogenic effects of tannic acid using the corneal pocket assay. While inhibition of corneal vessel growth was observed (data not shown), solution concentrations that inhibited corneal angiogenesis also resulted in animal weight loss, indicating that the observed reduction in angiogenesis is likely a result of compound toxicity, rather than a specific effect. Thus, we do not cite this data as supportive of tannic acid's ability to inhibit angiogenesis.

## Discussion

We have developed a high throughput screening assay to identify possible inhibitors of the interaction between PA and the CMG2 (ATR2) receptor. CMG2 has at least ten-fold higher affinity for PA than does TEM8 [33], is expressed alongside TEM8 in tumor cells, and has four-fold higher expression in endothelial cells [31] than is TEM8. Hence, while both TEM8 and CMG2 are of potential interest as targets for anti-angiogenic therapies, our first screens focused on CMG2. Data described here outlines the development and validation of a sensitive mix and measure homogeneous high throughput screening assay for inhibitors of PA-CMG2 interactions, based on FRET between dye-labeled proteins. Assay performance was characterized based on measured Z' values. A consistently high Z' value (Z'≥0.9) was observed for this high throughput screening assay, indicating exceptional performance. This assay is therefore highly sensitive to compounds that inhibit PA-CMG2 interactions and could be used to effectively generate lead compounds for cancer, other angiogenesis related diseases, and anthrax toxin therapies. In addition, small molecule inhibitors identified by this assay could potentially be used to investigate both the biological function of CMG2 and the molecular recognition processes operating at the cell surface.

Initial screening of a relatively small library resulted in identification of two compounds as inhibitors of the PA-CMG2 interaction. The first compound hit, cisplatin, has an established history as an anti-cancer agent and a wide range of documented physiological effects, largely mediated by DNA adduct formation. However, secondary binding analysis of cisplatin indicates that this compound inhibits PA-CMG2 binding by interaction with PA, as well as CMG2, and any observed anti-angiogenic effects caused by cisplatin administration may reflect mechanisms other than CMG2 binding, including formation of possible DNA adducts. The second hit, tannic acid, both binds CMG2 and inhibits angiogenesis as measured by endothelial cell migration. Tannic acid has multiple previously reported bioactivities including anti-angiogenic properties such as inhibition of cell migration and angiogenesis [63] and anti-cancer action in a variety of cancer cell [64] and tumor types [65]–[68]. Our identification of this tannic acid solution as a PA-CMG2 inhibitor suggests that one mechanism of tannic acid bioactivity may involve interactions with CMG2 that impact angiogenesis.

We cannot unequivocally conclude that the inhibitor isolated in this screen disrupts angiogenesis exclusively by interacting with CMG2. For example, we cannot rule out an anti-angiogenic effect exerted through a non-CMG2-mediated pathway, although its observed angiogenic activity is consistent with inhibition of CMG2. In addition, it is possible that the observed effects on angiogenesis result from cross-reactivity with TEM8. Both receptors are present in the corneal micropocket and endothelial cell migration assays, and given the relatively high sequence homology of TEM8 with CMG2, we suspect that some overlap in recognition is likely to occur. We are currently involved in additional high throughput screens to identify inhibitors of PA-TEM8 binding, and are interested to correlate results from these two studies.

Given its large range of bioactivities, tannic acid may not be a viable therapeutic lead. However, its identification in this screen is proof of principle that a simple, sensitive, and robust high throughput screening assay can identify compounds that inhibit the PA-CMG2 interaction and have potential anti-angiogenic properties. We anticipate that these observations will lay the groundwork for additional high throughput screens for PA-CMG2 inhibitors based on substantially larger libraries.

## Materials and Methods

### Protein expression, purification, and labeling

PA^E733C^ and CMG2^R40C C178A^ site-directed mutants were cloned, expressed in BL21 DE3 Star *E. coli* (Invitrogen), and purified using combinations of ion exchange (HP Q-Sepharose; GE Healthcare), affinity (GST Bind Agarose; Novagen), and size exclusion chromatography (Sephacryl 200HR; GE Healthcare) similar to those methods previously reported [58]. Protein purity was determined to be ≥85% by SDS-PAGE with Coomassie staining. These single cysteine mutants were labeled with either Alexa fluor 488 C_5_ maleimide, Alexa fluor 546 C_5_ maleimide, Alexa fluor 568 C_5_ maleimide, Alexa fluor 647 C_2_ maleimide (Invitrogen), or maleimide-PEG2-biotin (Pierce) using manufacturer recommended methods. The dye: protein ratios of all protein conjugates were determined by UV-VIS spectrophotometry or the HABA assay for biotin to be between 0.85 and 1.15. Protein activity for the FRET assay reagents was assessed using fluorescence spectroscopy to measure resonance energy transfer upon PA binding CMG2 *in vitro*.

### Fluorescence spectra and inhibitor IC_50_

All fluorescence spectra were acquired using a spectrofluorometer (QM-4; Photon Technology International) with a 75W Xe arc lamp excitation and photon counting photomultiplier detection. Slits for both the excitation and emission monochromators were set to achieve a 4 nm band pass. Briefly, fluorescence spectra 10 nM solutions of either PA^E733C*AF488^ or mixtures of PA^E733C*AF488^ and CMG2^R40C C178A*AF546^ in HEPES buffered saline + Tween-20 (HBST; 50 mM HEPES pH 7.4, 150 mM NaCl, 0.1 mM CaCl_2_, 0.1% Tween-20). Fluorescence emission spectra of the red-shifted versions of PA and CMG2 were measured in a similar fashion.

IC_50_ for both tannic acid and cisplatin was determined by addition of serial dilutions of the inhibitors in DMSO to a solution of CMG2^R40C C178A*AF546^ in HBST followed by addition of PA^E733C*AF488^ to a final concentration similar to that used in the screening assay (13 nM CMG2^R40C C178A*AF546^, 7.5 nM PA^E733C*AF488^) in 96-well-plates. Plates were incubated for 4 hours and read on a Genios (Tecan) plate reader with 485/10 nm excitation filter and 535/13 nm and 585/11 nm emission filters. The F585 nm/F535 nm fluorescence emission ratio was measured and plotted as a function of final inhibitor concentration. The cisplatin binding isotherm was fit to a single-site binding model using SigmaPlot (Systat).

### High throughput screening

For high throughput screening, 30 µl of a solution of 17 nM CMG2^R40C C178A*AF546^ in HBST was added to the wells of a barcode labeled Corning 3710 384-well-plate using a WellMate liquid handling robot (Matrix Technologies) with integrated stacker. Next, 0.3 µl of test compound (5 – 10 mg/mL) diluted in DMSO was added by pin transfer using a custom Epson robot to duplicate plates. Following a 1–3 hour incubation, 10 μl of a 30 nM PA^E733C*AF488^ solution in 50 mM HEPES pH 7.4, 150 mM NaCl, 0.1 mM CaCl_2_ was then added to all wells using the Wellmate and plates were incubated for 3–4 hours. Final CMG2 concentration (13 nM) and PA concentration (7.5 nM) were sufficient to promote quantitative binding of CMG2 in the absence of effective inhibitors, based on the previously reported K_d_ (≥170 pM) [58]. Incubation lengths varied between individual wells, as a function of the time required for delivery of library compounds to individual positions in the well-plate. Following incubation, plates were read on an Envision (PerkinElmer) plate reader using a 485/14 excitation filter, with 535/25 and 595/60 emission filters incorporating a barcode reader to correlate fluorescence measurements with plates. For each plate, 32 positive control wells were generated by adding 10mM EDTA to the CMG2 solution; 32 negative control wells were generated by addition of 10 mM NaCl to the CMG2 solution. Control wells did not receive addition of library compound(s).

### Endothelial cell migration assay

Human microvascular endothelial cells (Lonza) were maintained in EGM-2 media (Lonza) according to the vendor's instructions and used before passage 7. Polycarbonate transwell inserts, 6.5 mm diameter with 8.0 µm pores (Corning), were coated with 20µg/ml fibronectin (Sigma) overnight at 4°C. Cells were harvested and resuspended in EBM media (Lonza) containing 0.1% bovine serum albumin (Sigma). Cells (10,000 per well) were plated onto wells and placed within wells containing full serum EGM-2 medium alone or EGM-2 medium containing the molecule to be tested. Cells were allowed to migrate for 4 h with 5% CO_2_ at 37°C. Membranes were then rinsed once in PBS, and fixed and processed using Diff-Quick (Dade Diagnostics). Cells on the top of the membrane were removed using cotton-tipped applicators. Membranes were removed from the insert using a scalpel and mounted on slides, and the number of cells in 4 10× microscopic fields were counted.

### Endothelial cell proliferation assay

Human microvascular endothelial cells (HMVECs; Cambrex) were maintained in EGM-2 (Cambrex) according to the manufacturer's instructions, and used before passage 7. Proliferating cultures of cells were seeded at ∼10% confluence into 96 well plates. After attachment, medium was exchanged into that containing the designated concentration of inhibitor (Cambrex). Cells were allowed to grow for 24 and 72 h and then quantified using CyQUANT (Invitrogen) according to the manufacturer's protocols. The degree of proliferation in culture was measured by comparison of experimental wells with those fixed in absolute ethanol at t = 0.

### Mouse corneal micropocket assay

The corneal micropocket assay was performed as previously described [16], using pellets containing 80 ng of basic fibroblast growth factor (bFGF) in C57BL/6J mice. The treated groups received i.p. injections of compound in PBS. Treatment was started on the day after pellet implantation; control mice received vehicle alone i.p. The area of vascular response was assessed on the 5th postoperative day using a slit lamp. Typically, 10 eyes per group were measured.

### Surface plasmon resonance binding assay

Surface plasmon resonance (SPR) was used to determine binding of cisplatin and tannic acid to CMG2. CMG2^R40C R178A^ was labeled with biotin-PEG-maleimide (Pierce) for immobilization to a streptavidin-modified carboxydextran SPR sensor surface (SA; GE Healthcare). All experiments were performed using a Biacore X (GE Healthcare). Biotinylated CMG2 (100 nM in HBST) was flowed across channel 2 of the on the SA chip for 5 minutes at of 5 μl/min followed by a HBST wash. As a control, PEG-biotin was immobilized in channel 1 of the sensor chip under identical conditions. Solutions of tannic acid (1 μM) or cisplatin (500 μM) in HBST were flowed across the functionalized sensor chip at 10 μl/min and the sensorgrams recorded. Sensorgrams were recorded in triplicate.

### FRET kinetic assay

A FRET-based kinetic assay was used to monitor the rate of PA-CMG2 binding in the presence of cisplatin. First, fluorescently labeled PA^E733C*AF488^ (1 μM) or CMG2^R40C C178A*AF546^ (1 μM) were preincubated with 500 μM cisplatin in HBST for 30 minutes. This mixture was then rapidly diluted to a final concentration of 10 nM of each fluorescently labeled binding partner (CMG2 or PA respectively) in HBST and the donor and sensitized emission ratio was monitored over time. Control experiments consisted of preincubation with the DMSO vehicle alone.

### Ethical treatment of animals

This study was carried out in strict accordance with the recommendations in the Guide for the Care and Use of Laboratory Animals of the National Institutes of Health. All animal studies were conducted according to protocols approved by the Institutional Animal Care and Use Committee of Children's Hospital Boston (Protocol A06-10-086R). All surgery was performed under avertin anesthesia.

## References

[1] Folkman J, Klagsbrun M (1987). Angiogenic factors.. Science.

[2] Engerman RL, Pfaffenbach D, Davis MD (1967). Cell turnover of capillaries.. Lab Invest.

[3] Poole TJ, Coffin JD (1989). Vasculogenesis and angiogenesis: Two distinct morphogenetic mechanisms establish embryonic vascular pattern.. J Exp Zool.

[4] Reynolds LP, Killilea SD, Redmer DA (1992). Angiogenesis in the female reproductive system.. Faseb J.

[5] Folkman J (2007). Angiogenesis: An organizing principle for drug discovery?. Nat Rev Drug Discov.

[6] Folkman J (1975). Tumor angiogenesis: A possible control point in tumor growth.. Ann Intern Med.

[7] Folkman J, Watson K, Ingber D, Hanahan D (1989). Induction of angiogenesis during the transition from hyperplasia to neoplasia.. Nature.

[8] Rogers MS, D'Amato RJ (2006). The effect of genetic diversity on angiogenesis.. Exp Cell Res.

[9] D'Amato R, Adamis AP (1995). Angiogenesis inhibition in age-related macular degeneration.. Ophthalmology.

[10] Michaelson IC (1948). The mode of development of the vascular system of the retina, with some observations on its significance for certain retinal disease.. Trans Ophthalmol Soc U K.

[11] Adamis AP, Aiello LP, D'Amato R (1999). Angiogenesis and ophthalmic disease.. Angiogenesis.

[12] Singhal S, Mehta J, Desikan R, Ayers D, Roberson P (1999). Antitumor activity of thalidomide in refractory multiple myeloma.. N Engl J Med.

[13] Gragoudas ES, Adamis AP, Cunningham ET, Feinsod M, Guyer DR (2004). Pegaptanib for neovascular age-related macular degeneration.. N Engl J Med.

[14] Hurwitz H, Fehrenbacher L, Novotny W, Cartwright T, Hainsworth J (2004). Bevacizumab plus irinotecan, fluorouracil, and leucovorin for metastatic colorectal cancer.. N Engl J Med.

[15] Rosenfeld PJ, Brown DM, Heier JS, Boyer DS, Kaiser PK (2006). Ranibizumab for neovascular age-related macular degeneration.. N Engl J Med.

[16] Rogers MS, Christensen KA, Birsner AE, Short SM, Wigelsworth DJ (2007). Mutant anthrax toxin B moiety (protective antigen) inhibits angiogenesis and tumor growth.. Cancer Res.

[17] Gordon VM, Klimpel KR, Arora N, Henderson MA, Leppla SH (1995). Proteolytic activation of bacterial toxins by eukaryotic cells is performed by furin and by additional cellular proteases.. Infect Immun.

[18] Bradley KA, Mogridge J, Mourez M, Collier RJ, Young JA (2001). Identification of the cellular receptor for anthrax toxin.. Nature.

[19] Scobie HM, Rainey GJ, Bradley KA, Young JA (2003). Human capillary morphogenesis protein 2 functions as an anthrax toxin receptor.. Proc Natl Acad Sci U S A.

[20] St Croix B, Rago C, Velculescu V, Traverso G, Romans KE (2000). Genes expressed in human tumor endothelium.. Science.

[21] Nanda A, St Croix B (2004). Tumor endothelial markers: New targets for cancer therapy.. Curr Opin Oncol.

[22] Davies G, Rmali KA, Watkins G, Mansel RE, Mason MD (2006). Elevated levels of tumour endothelial marker-8 in human breast cancer and its clinical significance.. Int J Oncol.

[23] Cullen M, Seaman S, Chaudhary A, Yang MY, Hilton MB (2009). Host-derived tumor endothelial marker 8 promotes the growth of melanoma.. Cancer Res.

[24] Rmali KA, Watkins G, Harrison G, Parr C, Puntis MC (2004). Tumour endothelial marker 8 (TEM-8) in human colon cancer and its association with tumour progression.. Eur J Surg Oncol.

[25] Rmali KA, Puntis MC, Jiang WG (2005). Prognostic values of tumor endothelial markers in patients with colorectal cancer.. World J Gastroenterol.

[26] Rmali KA, Puntis MC, Jiang WG (2005). TEM-8 and tubule formation in endothelial cells, its potential role of its vW/TM domains.. Biochem Biophys Res Commun.

[27] Nanda A, Carson-Walter E, Seaman S, Barber TD, Stampfl J (2004). TEM8 interacts with the cleaved C5 domain of collagen alpha 3(VI).. Cancer Res.

[28] Werner E, Kowalczyk AP, Faundez V (2006). Anthrax toxin receptor 1/tumor endothelium marker 8 mediates cell spreading by coupling extracellular ligands to the actin cytoskeleton.. J Biol Chem.

[29] Chaudhary A, Hilton MB, Seaman S, Haines DC, Stevenson S (2012). TEM8/ANTXR1 blockade inhibits pathological angiogenesis and potentiates tumoricidal responses against multiple cancer types.. Cancer Cell.

[30] Bell SE, Mavila A, Salazar R, Bayless KJ, Kanagala S (2001). Differential gene expression during capillary morphogenesis in 3D collagen matrices: Regulated expression of genes involved in basement membrane matrix assembly, cell cycle progression, cellular differentiation and G-protein signaling.. J Cell Sci.

[31] Reeves CV, Dufraine J, Young JA, Kitajewski J (2010). Anthrax toxin receptor 2 is expressed in murine and tumor vasculature and functions in endothelial proliferation and morphogenesis.. Oncogene.

[32] Dowling O, Difeo A, Ramirez MC, Tukel T, Narla G (2003). Mutations in capillary morphogenesis gene-2 result in the allelic disorders juvenile hyaline fibromatosis and infantile systemic hyalinosis.. Am J Hum Genet.

[33] Liu S, Crown D, Miller-Randolph S, Moayeri M, Wang H (2009). Capillary morphogenesis protein-2 is the major receptor mediating lethality of anthrax toxin in vivo.. Proc Natl Acad Sci U S A.

[34] Reeves CV, Wang X, Charles-Horvath PC, Vink JY, Borisenko VY (2012). Anthrax toxin receptor 2 functions in ECM homeostasis of the murine reproductive tract and promotes MMP activity.. PLoS One.

[35] Peters DE, Zhang Y, Molinolo AA, Miller-Randolph S, Szabo R (2012). Capillary morphogenesis protein-2 is required for mouse parturition by maintaining uterine collagen homeostasis.. Biochem Biophys Res Commun.

[36] Mock M, Mignot T (2003). Anthrax toxins and the host: A story of intimacy.. Cell Microbiol.

[37] Mogridge J, Cunningham K, Lacy DB, Mourez M, Collier RJ (2002). The lethal and edema factors of anthrax toxin bind only to oligomeric forms of the protective antigen.. Proceedings of the National Academy of Sciences of the United States of America.

[38] Abrami L, Liu S, Cosson P, Leppla SH, van dG (2003). Anthrax toxin triggers endocytosis of its receptor via a lipid raft-mediated clathrin-dependent process.. J Cell Biol.

[39] Collier RJ, Young JA (2003). Anthrax toxin.. Annu Rev Cell Dev Biol.

[40] Liu S, Leppla SH (2003). Cell surface tumor endothelium marker 8 cytoplasmic tail-independent anthrax toxin binding, proteolytic processing, oligomer formation, and internalization.. J Biol Chem.

[41] Wesche J, Elliott JL, Falnes PO, Olsnes S, Collier RJ (1998). Characterization of membrane translocation by anthrax protective antigen.. Biochemistry.

[42] Mourez M, Kane RS, Mogridge J, Metallo S, Deschatelets P (2001). Designing a polyvalent inhibitor of anthrax toxin.. Nat Biotechnol.

[43] Sellman BR, Mourez M, Collier RJ (2001). Dominant-negative mutants of a toxin subunit: An approach to therapy of anthrax.. Science.

[44] Karginov VA, Robinson TM, Riemenschneider J, Golding B, Kennedy M (2004). Treatment of anthrax infection with combination of ciprofloxacin and antibodies to protective antigen of bacillus anthracis.. FEMS Immunol Med Microbiol.

[45] Aulinger BA, Roehrl MH, Mekalanos JJ, Collier RJ, Wang JY (2005). Combining anthrax vaccine and therapy: A dominant-negative inhibitor of anthrax toxin is also a potent and safe immunogen for vaccines.. Infect Immun.

[46] Cui X, Li Y, Moayeri M, Choi GH, Subramanian GM (2005). Late treatment with a protective antigen-directed monoclonal antibody improves hemodynamic function and survival in a lethal toxin-infused rat model of anthrax sepsis.. J Infect Dis.

[47] Batty S, Chow EM, Kassam A, Der SD, Mogridge J (2006). Inhibition of mitogen-activated protein kinase signalling by bacillus anthracis lethal toxin causes destabilization of interleukin-8 mRNA.. Cell Microbiol.

[48] Galoyan AA, Grigoryan SL, Badalyan KV (2006). Treatment and prophylaxis of anthrax by new neurosecretory cytokines.. Neurochem Res.

[49] Gubbins MJ, Berry JD, Corbett CR, Mogridge J, Yuan XY (2006). Production and characterization of neutralizing monoclonal antibodies that recognize an epitope in domain 2 of bacillus anthracis protective antigen.. FEMS Immunol Med Microbiol.

[50] Gujraty KV, Joshi A, Saraph A, Poon V, Mogridge J (2006). Synthesis of polyvalent inhibitors of controlled molecular weight: Structure-activity relationship for inhibitors of anthrax toxin.. Biomacromolecules.

[51] Vitale L, Blanset D, Lowy I, O'Neill T, Goldstein J (2006). Prophylaxis and therapy of inhalational anthrax by a novel monoclonal antibody to protective antigen that mimics vaccine-induced immunity.. Infect Immun.

[52] Xiong Y, Wiltsie J, Woods A, Guo J, Pivnichny JV (2006). The discovery of a potent and selective lethal factor inhibitor for adjunct therapy of anthrax infection.. Bioorg Med Chem Lett.

[53] Alvarez Z, Abel-Santos E (2007). Potential use of inhibitors of bacteria spore germination in the prophylactic treatment of anthrax and clostridium difficile-associated disease.. Expert Rev Anti Infect Ther.

[54] Scorpio A, Tobery SA, Ribot WJ, Friedlander AM (2008). Treatment of experimental anthrax with recombinant capsule depolymerase.. Antimicrob Agents Chemother.

[55] Vuyisich M, Gnanakaran S, Lovchik JA, Lyons CR, Gupta G (2008). A dual-purpose protein ligand for effective therapy and sensitive diagnosis of anthrax.. Protein J.

[56] Schneemann A, Manchester M (2009). Anti-toxin antibodies in prophylaxis and treatment of inhalation anthrax.. Future Microbiol.

[57] Basha S, Rai P, Poon V, Saraph A, Gujraty K (2006). Polyvalent inhibitors of anthrax toxin that target host receptors.. Proc Natl Acad Sci U S A.

[58] Wigelsworth DJ, Krantz BA, Christensen KA, Lacy DB, Juris SJ (2004). Binding stoichiometry and kinetics of the interaction of a human anthrax toxin receptor, CMG2, with protective antigen.. J Biol Chem.

[59] Zhang J, Chung TDY, Oldenburg KR (1999). A simple statistical parameter for use in evaluation and validation of high throughput screening assays.. Journal of Biomolecular Screening.

[60] McGovern SL, Helfand BT, Feng B, Shoichet BK (2003). A specific mechanism of nonspecific inhibition.. J Med Chem.

[61] Bijman MN, van NA, Laurens N, van Hinsbergh V, Boven E (2006). Microtubule-targeting agents inhibit angiogenesis at subtoxic concentrations, a process associated with inhibition of Rac1 and Cdc42 activity and changes in the endothelial cytoskeleton.. Mol Cancer Ther.

[62] Moayeri M, Wiggins JF, Lindeman RE, Leppla SH (2006). Cisplatin inhibition of anthrax lethal toxin.. Antimicrob Agents Chemother.

[63] Chen X, Beutler JA, McCloud TG, Loehfelm A, Yang L (2003). Tannic acid is an inhibitor of CXCL12 (SDF-1alpha)/CXCR4 with antiangiogenic activity.. Clin Cancer Res.

[64] Kamei H, Koide T, Hashimoto Y, Kojima T, Hasegawa M (1999). Tumor cell growth suppression by tannic acid.. Cancer Biother Radiopharm.

[65] Mukhtar H, Das M, Khan WA, Wang ZY, Bik DP (1988). Exceptional activity of tannic acid among naturally occurring plant phenols in protecting against 7,12-dimethylbenz(a)anthracene-, benzo(a)pyrene-, 3-methylcholanthrene-, and N-methyl-N-nitrosourea-induced skin tumorigenesis in mice.. Cancer Res.

[66] Das M, Bickers DR, Mukhtar H (1989). Protection against chemically induced skin tumorigenesis in SENCAR mice by tannic acid.. Int J Cancer.

[67] Koide T, Kamei H, Hashimoto Y, Kojima T, Hasegawa M (1999). Tannic acid raises survival rate of mice bearing syngeneic tumors.. Cancer Biother Radiopharm.

[68] Marienfeld C, Tadlock L, Yamagiwa Y, Patel T (2003). Inhibition of cholangiocarcinoma growth by tannic acid.. Hepatology.

